# The Canadian Open Neuroscience Platform—An open science framework for the neuroscience community

**DOI:** 10.1371/journal.pcbi.1011230

**Published:** 2023-07-27

**Authors:** Rachel J. Harding, Patrick Bermudez, Alexander Bernier, Michael Beauvais, Pierre Bellec, Sean Hill, Agâh Karakuzu, Bartha M. Knoppers, Paul Pavlidis, Jean-Baptiste Poline, Jane Roskams, Nikola Stikov, Jessica Stone, Stephen Strother, CONP Consortium, Alan C. Evans

**Affiliations:** 1 Structural Genomics Consortium, University of Toronto, Toronto, Ontario, Canada; 2 McGill Centre for Integrative Neuroscience, Montreal Neurological Institute, McGill University, Montréal, Québec, Canada; 3 Centre of Genomics and Policy, Department of Human Genetics, Faculty of Medicine, McGill University, Montréal, Québec, Canada; 4 Centre de Recherche de l’Institut Universitaire de Gériatrie de Montréal, Montréal, Québec, Canada; 5 Department of Psychology, Université de Montréal, Montréal, Québec, Canada; 6 Krembil Centre for Neuroinformatics, Centre for Addiction and Mental Health, Toronto, Ontario, Canada; 7 Institute of Medical Sciences, University of Toronto, Toronto, Ontario, Canada; 8 Department of Psychiatry, University of Toronto, Toronto, Ontario, Canada; 9 Department of Physiology, University of Toronto, Toronto, Ontario, Canada; 10 Canada Research Chair in Law and Medicine, Montréal, Québec, Canada; 11 Michael Smith Laboratories, University of British Columbia, Vancouver, British Columbia, Canada; 12 Department of Psychiatry, University of British Columbia, Vancouver, British Columbia, Canada; 13 Djavad Mowafaghian Center for Brain Health, University of British Columbia, Vancouver, British Columbia, Canada; 14 ORIGAMI Neuro Data Science Laboratory, Montreal Neurological Institute, McGill University, Montréal, Québec, Canada; 15 Neurosurgery University of Washington, Seattle, Washington, United States of America; 16 NeuroPoly Lab, Institute of Biomedical Engineering, Polytechnique Montréal, Montréal, Québec, Canada; 17 Montréal Heart Institute, Université de Montréal, Montréal, Québec, Canada; 18 Center for Advanced Interdisciplinary Research, Ss. Cyril and Methodius University, Skopje, North Macedonia; 19 Rotman Research Institute, Baycrest, and Department of Medical Biophysics, University of Toronto, Toronto, Ontario, Canada; University of Virginia, UNITED STATES

## Abstract

The Canadian Open Neuroscience Platform (CONP) takes a multifaceted approach to enabling open neuroscience, aiming to make research, data, and tools accessible to everyone, with the ultimate objective of accelerating discovery. Its core infrastructure is the CONP Portal, a repository with a decentralized design, where datasets and analysis tools across disparate platforms can be browsed, searched, accessed, and shared in accordance with FAIR principles. Another key piece of CONP infrastructure is NeuroLibre, a preprint server capable of creating and hosting executable and fully reproducible scientific publications that embed text, figures, and code. As part of its holistic approach, the CONP has also constructed frameworks and guidance for ethics and data governance, provided support and developed resources to help train the next generation of neuroscientists, and has fostered and grown an engaged community through outreach and communications. In this manuscript, we provide a high-level overview of this multipronged platform and its vision of lowering the barriers to the practice of open neuroscience and yielding the associated benefits for both individual researchers and the wider community.

## Introduction

Neurological diseases represent an increasing burden to societies globally [1,2] and this trend will only be exacerbated as demographics shift with aging populations. The problem is compounded for many common neurological and neuropsychiatric disorders, as our current understanding of ‘normal’ brain function and how it is modulated in disease is lacking and few disease-modifying therapies are available [3]. Major contributors to our ignorance of the mechanisms of brain function and dysfunction are insufficient statistical power and a lack transparent data processing pipelines. Too many underpowered studies, in which inaccessible data are analyzed in non-reproducible or black-box pipelines, often lead to contradictory findings that undermine the search for reliable insight into brain processes. Open science practices offer a means to share data and tools such that we can build sufficiently large and representative datasets to address questions that cannot be answered by traditional approaches.

Open science is an umbrella term that covers the open dissemination of data, software, materials, methodologies, manuscripts, and other products of scientific research, making the entire process more transparent, robust, and accessible. Open science is widely considered to be beneficial for both scientists [4] and patients [5], and it is strongly supported by the public at large [6] and a broad cross-section of international entities including UNESCO [7], WHO [8], the National Institutes of Health, and multiple levels of the Canadian government [9]. The growing open neuroscience movement strives to create stronger systems to share data and reproducible methods freely and responsibly among brain scientists, thereby accelerating discovery in the study of normal brain function and the pathophysiological mechanisms of disease and expediting translation of new knowledge into the clinic as diagnostics, therapeutics, and cures. Large-scale challenges faced by neuroscientists, such as reproducibility and data reuse, can be improved through open science practices. However, widespread adoption in the field has yet to be achieved and open science initiatives are often fragmented and short-lived.

There are formidable challenges to combining different data modalities within the complex data-science strategies increasingly used in both basic and clinical neuroscience. A multidisciplinary response is required, one aspect of which is effective open science methods and data sharing [10]. Most patients and patient advocates want their data (suitably anonymized) to be made more widely accessible, so as to accelerate the pace of discovery of earlier diagnostics and more effective, personalized therapeutic interventions [11]. However, narrowly conceived privacy constraints, fear of legal exposure, and academic protectionism are opposing considerations that must be managed and reformed through active cross-discipline and cross-sector collaboration. The Canadian Open Neuroscience Platform (CONP) is focused on developing and disseminating stronger open science practices, from data sharing to manuscript publication, as well as making neuroscience research accessible to a broader community, within Canada and beyond. Here, we present the mission of the CONP as well as an overview of the resources and infrastructure we have built and disseminated to reduce the technical, administrative, and educational barriers to the practice of open neuroscience.

## An overview of the CONP: A pan-Canadian initiative to accelerate discovery in brain research

The CONP was established as a national network of neuroscience research centers collaborating on a series of new open neuroscience initiatives designed to provide functions and services only partially met in disparate existing efforts. Specifically, the CONP has constructed infrastructure and resources that support (i) free sharing of multimodal neuroscience research data and analytic tools, (ii) cross-disciplinary training for young scientists wishing to operate at the interfaces of neuroimaging, behavioral neuroscience, genetics, and data science, (iii) creation of policy frameworks that embed these technical capabilities within an ethically sound model of data governance and dissemination, (iv) the growth of open publishing, such that published results can be made available for secondary analysis by the wider scientific community, and (v) building partnerships with international peer initiatives that espouse similar open science principles. The CONP is operated through a collection of committees, each developing and overseeing specific aspects of the platform. These interact with and report to a Steering committee, which sets the strategic priorities for the domain-specific committees and oversees the operational activity of the CONP network. Members of the various committees are drawn from major partner universities across the network and represent a broad cross-section of neuroscientific specialties.

## The CONP Portal—A decentralized neuroscience sharing platform to facilitate access and reduce data silos

The CONP Portal provides researchers with access to open neuroscience datasets and the capacity to share their own data, as well as obtain an array of analysis tools that can be applied to data either locally, through remote high-performance computing (HPC) resources, or via cloud services [12]. The Portal currently hosts more than 90 tools and 70 datasets and caters to a significant range of use cases in open neuroscience research, acting to reduce ‘research silos’ by integrating across major international open science sharing infrastructures as well as Canadian data repositories. The Portal can provide users with simplified access to data from several domain-agnostic datastores (e.g., OSF, Zenodo) and data-management systems such as LORIS [13,14], XNAT [15], and Brain-CODE [16]. Through its adherence to the FAIR principles of data- and tool-sharing [10], the Portal offers rigorous and reproducible paths to both big-data researchers who require massively parallel processing through HPC/cloud services and those who process relatively small datasets locally on a single computer. The Portal’s foundation upon open standards, flexibility of interface (e.g., command line versus web browser), and lowering of technical overhead and investment costs allows it to be integrated into a wide variety of research scenarios and workflows for diverse neuroscience applications. The Portal is detailed in Poline and colleagues [12] and its source code is fully open and available on GitHub under an MIT license.

### Datasets—Flexible hosting and access with cross-database search

The CONP Portal provides one-stop access to data residing on different infrastructures through its flexible distributed management system (**Table 1**). This decentralized, federating design provides search capabilities that operate across available datasets and analysis tools (irrespective of the infrastructure on which they reside), thereby increasing discoverability, FAIRness, and likelihood of being scientifically exploited. It also provides researchers with the ability to find and retrieve scientifically valuable conjunctions of data across otherwise siloed datasets, agglomerate smaller datasets into supersets for increased statistical power and potential for discovery, and gain access to HPC resources and containerized analysis tools. Users who wish to include their data in the Portal can choose among multiple pathways, including storage provided by the OSF, Zenodo, or the CONP, or benefit from greater flexibility still in data-hosting location through the combined use of the DataLad distributed data management system [17] and the GitHub open software repository to host the dataset metadata.

**Table 1:** Overview of commonly used data repositories in the neuroscience community and their features.

Platform	Storage model	Research focus	Access control	Tool-data integration	Dataset browsing across infrastructures	Dataset retrieval across infrastructures
CONP Portal	Decentralized	Neuroscience	Determined by storage provider	Boutiques/CBRAIN	Yes	Yes
Zenodo	Centralized	General	Public or restricted	None	No	No
OSF	Centralized	General	Public or private	None	No	No
NeuroMorpho	Centralized	Digital neurons	Public	None	No	No
OpenNeuro	Centralized	BIDS imaging datasets	Public after embargo	BIDS Apps	No	No
NIMH Data Archive	Centralized	Human subjects	Restricted	None	No	No
FRDR	Centralized	General (Canada only)	Public after embargo	None	Yes	No
Harvard Dataverse	Centralized	General	Public with Restricted subsets	None	No	No
EBRAINS	Centralized	General	Public or Restricted	Simulation tools	No	No
NIF	Centralized	General	Public	None	No	No
NITRC	Centralized	Neuroimaging	Public	>500 tools available	No	Yes
BrainLife	Centralized	Imaging	Public	Specific Apps	No	No

Datasets shared through the Portal are annotated using the Data Tags Suite model [18] to ensure uniform, extensible, and rich descriptions of data and their provenance, essential features of metadata for adherence to the FAIR principles. It contains obligatory fields (e.g., dataset description, names and affiliations, the license under which a dataset is released, keywords, data types/formats, and ethics information, where applicable), optional fields (e.g., associated publications, funding bodies, and cross-referencing–derived datasets), and fields added for the CONP Portal to expand provenance information and searchability (e.g., a structured record of the dataset’s source). The CONP Portal also leverages other open-source technologies such as CBRAIN [19,20], which allows users to run analysis pipelines on a network of HPC clusters through a friendly web-browser–based interface, Boutiques [21], a workflow containerization standard that enables the publishing and reproducible execution of software on a variety of platforms and computing architectures, and LORIS [13,22,23], a web-based, patient-centric, data and project management system capable of hosting longitudinal behavioral, clinical, neuroimaging, and genetic data.

A search function allows users to easily find datasets by keyword and, subsequently, download them through either a one-click, direct download function or through DataLad. The latter allows for the download of a large number of files selected from different datasets (irrespective of source infrastructure) and the integration of this ‘data fetching’ ability into automated processing pipelines. Furthermore, different access tiers can be accommodated, depending on ethical or legal requirements, without compromising a dataset’s discoverability within the Portal. Fully open datasets with no access limitation (currently approximately 85% of the Portal’s datasets) can be downloaded directly through a web browser, while the DataLad backend adds a command line, file level download option. Currently, the CONP Portal is one of the only decentralized neuroscience repositories that not only indexes remote datasets but also enables downloading data from these datasets directly through a browser interface or via Datalad.

The harmonization of metadata across datasets and tools in the Portal continues and the CONP’s Communications Committee works in partnership with the Technical Committee to help evaluate data and tool submissions with respect to their adherence to FAIR principles. For example, where possible, descriptions are assessed for their clarity and accessibility through an iterative editorial process in collaboration with data owners/maintainers.

### Analysis tools and data-processing—Reproducible analyses from laptop to supercomputer

The CONP Portal provides access to a wide range of analysis tools and pipelines, applicable to a variety of different data types (neuroimaging, electrophysiological, genomic, etc.). These tools are packaged with the Boutiques containerization standard [21], allowing for reproducible execution of software on a variety of platforms, both local and distributed. Boutiques descriptors link to Docker or Singularity container images with all their dependencies installed and configured for execution, including workflow engines such as TIGR-PURR [24] or web platforms such as CBRAIN [20] and VIP [25]. For use cases that benefit from additional computational resources, the Clowdr command-line tool and Python API [26] can be used to execute CONP Portal tools on clusters like those of the Digital Research Alliance of Canada or Amazon Elastic Computing Cloud. The Boutiques Python API enables tool integration into external pipeline engines, e.g., Pydra, Nextflow, and Apache Spark, and Boutiques-packaged tools are also amenable to local processing on standalone PCs or lab servers, thereby serving the full range of computing power.

### CONP Portal workflow possibilities—Discovery, access, and processing flexibility

The CONP Portal helps researchers discover multiple sources of data responding to given search criteria (e.g., according to modality, participant population), retrieve those data either through the web interface or through command-line access that can be automated and integrated into an analysis pipeline, and then apply tools either locally or through Cloud computing. These features also serve as motivation for a researcher to share their data through the CONP Portal, given the attractiveness of increased visibility alongside other datasets irrespective of source and facilitated access to HPC.

The CONP’s mission to continually develop interoperability of existing data infrastructures is crucial to reducing waste and underexplored, isolated datasets. It is not technically or financially feasible for the developers of a data-sharing platform to create customized interoperability between themselves and each of their peer systems. Much more economically and powerfully, the CONP Portal acts as an interoperability hub such that each data platform can adopt a single compatibility layer based on DataLad open standards, which, through the CONP Portal, will make their data equally and mutually visible. The CONP Technical Committee continues to enhance its already flexible API and standardization to facilitate access to data from other impactful data-sharing platforms.

## NeuroLibre is the first preprint server for fully reproducible analyses in neuroscience

Recently developed open science alternatives to traditional manuscript formats offer the means for fully reproducible workflows to address difficulties including incomplete documentation, obsolescence of dependencies, missing reproducibility assets, or version mismatch between the code that has been shared and the code used in the article. For example, Jupyter books [27] can be used to make computational notebooks and web-native documents that embed scientific text together with the code generating the scientific figures and analyses. Jupyter notebooks are a powerful research tool but, until now, they have not been capitalized upon by a comprehensive publishing platform that ensures all necessary assets for reproducibility are properly listed, accessible, and archived for long-term preservation (crucial for reproducibility and avoidance of ‘software rot’), tested, and made executable.

NeuroLibre is an executable preprint server that packages the necessary code, data, and execution environment for reproducible analyses in neuroscience, thereby serving as the first venue for the publication, interactive readership, and full reproducibility of Jupyter books [28]. Beyond simply serving Jupyter notebooks, NeuroLibre implements a standard for the minimum requirements to generate publishable research objects that integrate scientific narratives, figures, and code with the full computational environment required to run that code (e.g., software, data). Reproducing an analysis or iterative variations of an analysis can be easily achieved through a web browser session that spawns a personalized instance of the computational environment and data that a user can modify and execute, as demonstrated in the executable preprints published to date [29,30]. Collectively, NeuroLibre’s features augment and democratize the scientific community’s ability to perform deep and comprehensive peer review, with all its concomitant benefits.

**Fig 1:**
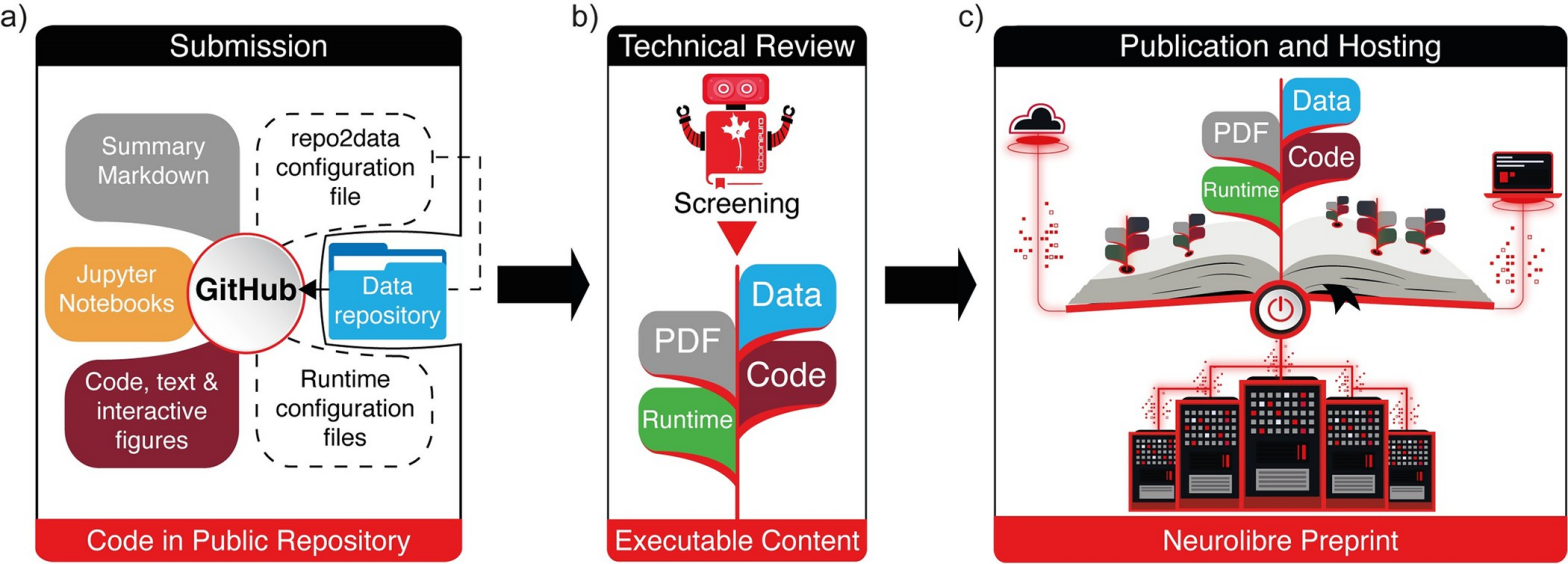
Workflow for submitting and publishing a NeuroLibre preprint. (a) The source files of a NeuroLibre preprint, including a markdown file in a public code repository (such as GitHub or GitLab) containing a high-level summary, a list of authors and affiliations, and a set of configuration files for declaring data and runtime dependencies for the executable content of the preprint. (b) Upon submission to NeuroLibre, a technical review is completed on GitHub using an editorial bot (RoboNeuro) to ensure the functionality of the preprint. (c) All the outputs associated with an accepted submission (Jupyter Book, generated PDF summary, Docker image and data) are assigned a DOI and transferred to the NeuroLibre production server for hosting.

In essence, NeuroLibre is a publication workflow that can satisfy the content moderation and technical review required for a public code repository (**Fig 1**). This is not a peer review of the subject-specific material but rather a technical review to verify a notebook’s scope and to ensure it is operational once published on the platform. To achieve this, NeuroLibre has developed two dedicated full-stack servers (one for preview and one for the preprint stage) with API endpoints that process submissions to the platform as follows. First, the platform creates a reproducible runtime and then executes all the computational Markdown files in it by using the data provided by the authors. This process can be triggered upon each change made to the authors’ preprint repository. In the event of a build failure, log files report the issue, while a successful build results in the creation of a Docker image and a Jupyter Book for the corresponding commit hash. The API endpoint providing this functionality can be accessed by authors to test their repositories and given a technical screening from GitHub issues. Once the build succeeds, the publication-ready reproducibility assets (all the repository content, Docker image, data, built Jupyter Book, and the PDF) are moved to the production server. Except for the PDF, all reproducibility assets are archived on Zenodo and assigned a DOI that is informed by the git commit reference from which they were built. These DOIs are minted for the meta-summary PDF that is published as a preprint. After publication, the static HTML content of the Jupyter Book is served using a high-performance web server that uses a private BinderHub instance whenever a reader requests an online code execution session to reproduce the analyses.

### NeuroLibre offers new possibilities for collaboration and partnership with journals and the publishing industry

Several proof-of-concept notebooks detailing analyses of neuroscience datasets have been published with NeuroLibre and traditional academic publishers concurrently, as was the case for a recent tutorial on T1 mapping [31]. This tutorial is distributed under a Creative Commons license, making it possible to access and interact with the associated text, code, and data, and thereby act as a powerful complement to the traditionally published book chapter in which the main text is published. In a further example, another NeuroLibre research object acts as a companion to a GigaScience article [32], providing synthetic data to reproduce the publication’s machine learning experiments without sharing the confidential data it used. Finally, the journal *eLife* recently published an interactive meta-analysis on myelin imaging with MRI [30], which was first available to the community as a NeuroLibre preprint. The meta-analysis contained within this work is also available as an executable research article and its public domain template can be adapted to other systematic reviews. The article has received more than 100 citations since publication, indicating the interest and utility of NeuroLibre within the neuroscience research landscape.

Though currently in beta testing, with a view to open to public submissions in December 2023, NeuroLibre has already been featured in a few journal editorials [33–36]. Following the standards defined by NeuroLibre, executable notebooks for some of these journals were built by a small team of collaborators and have been featured in the recently announced MRPub [37], as well as in a survey on code sharing conducted by *PLoS* [38]. The next step to advance the NeuroLibre mission is to expand its partnerships with journals and educational initiatives [39]. Several journals have expressed interest in piloting a formal peer-review of reproducible preprints hosted in NeuroLibre and NeuroLibre was also used as part of educational courses held at the 2021 meeting of the Organization for Human Brain Mapping.

NeuroLibre is built with open-source components and features detailed developer documentation, so that it can be freely adapted to meet the needs of other communities outside neuroscience. The development of NeuroLibre has benefited from several high-quality open-source projects, namely Jupyter Book [40], Jupyter Hub, and the publication workflow developed by the Journal of Open Source Software, and the NeuroLibre team, in turn, is contributing development and documentation back to these community-driven projects. As the scientific community and funding agencies increasingly require that data and analysis strategies be transparent and openly accessible to the community, the NeuroLibre model for Open Publishing (along with the CONP’s infrastructure for data and tool sharing) provides a framework to meet these requirements. As it is currently available to the research community at no cost, NeuroLibre also aims to help researchers adhere to the aforementioned requirements for dissemination of research results without additional financial burden.

## Ethics and data governance frameworks crafted for sharing neuroscience data in Canada

In recognition of the central role that ethics must play in the implementation of open neuroscience, the CONP has crafted policies and governance guidance for the responsible, open sharing of neuroscience data [41]. These resources are underpinned by three overarching goals: protecting participant autonomy, promoting trust, and realizing the human right to benefit from scientific advancement [42].

First, the “Ethics and Data Governance Framework” [43] provides a comprehensive overview of the stakeholders in neuroscience studies, including participants, researchers, and the public, and underscores the need for everyone to benefit from responsibly conducted science [44]. In service of these objectives, the Framework provides guidance on issues such as autonomy, privacy and security, conditions of data access, capacity to consent, and community engagement.

Second, the “Publication and Commercialization Policies” [45] provide ‘best practices’ concerning open-access journals, the posting of either preprints or postprints, and the open sharing of datasets, software, and other research outputs. The overarching ethos of the policy is that scientific outputs should be open unless there is a compelling justification for not doing so, e.g., a risk to participant privacy in line with recent international approaches to open science [7]. It also examines the complex relationship between open science and IP rights [46] and, while underscoring the need for proper attribution in all outputs, emphasizes openness, strongly discourages obtention of IP protection of shared resources, and advises that free and open licenses be used wherever possible, including for derivative works.

Finally, the CONP has developed an “ethics toolkit,” [47] a set of practical resources to assist students, researchers, and scientists in the necessary steps towards making their work as open as possible. The toolkit presents a cohesive array of resources to further the practice of open science while preserving the flexibility required by individuals within the community with respect to the degree of openness appropriate to each research program and dataset. It helps researchers identify key elements in the design of their projects that are often required for the open sharing of neuroscience data, such as model consent language, approaches to de-identification, and a retrospective consent filter [48].

## Training the next generation neuroscientists at research institutions across Canada

The CONP supports the next generation of open neuroscientists through its Scholar Program, a competitive award process that funds highly qualified applicants to pursue collaborative projects across disciplines and institutions. These scholars develop platforms, resources, datasets, analytics, and other resources that contribute to the advancement of open neuroscience. More than 60 trainees have served as CONP scholars to date, producing innovative research that has fed back into the ongoing development of the CONP and the broader international neuroscience community via the CONP Portal and other avenues. The CONP scholar community participates in webinars and educational activities to expose them to the core components of the CONP and to promote their active engagement in the CONP community and the practice of open neuroscience more widely. CONP scholars thus serve as open neuroscience ‘ambassadors,’ reinvesting their knowledge into developing CONP resources and into the promotion of open workflows among their peers.

The CONP has also partnered with the Training Space of the International Neuroinformatics Coordinating Facility (INCF, [49]) to share training materials and expertise from its network more broadly and help expand the INCF’s training resources, which incorporates educational content from all over the world (e.g., NeuroMatch Academy [50], The Virtual Brain [51], NeuroData Without Borders [52]). CONP members and scholars have contributed materials and curation to develop thematically coherent “Study Tracks.” One of these, an “Open Neuroscience Starter Kit,” aims to help trainees navigate through the ever-expanding environment of tools and skills required for open neuroscience workflows (from “open-by-design” experimental conception, through reproducible analysis, to safe data sharing and open publishing).

## Lessons learned and challenges for the CONP

The CONP still faces many challenges common to the open science movement. The COVID-19 pandemic has increased favorable outlooks on open science, data sharing, and publishing practices in the biomedical field that enable rapid, robust, and reproducible science [53], but there is still resistance within the community to adopt and implement fully open science workflows. Despite appetite to change the status quo, many researchers are subject to environmental pressures and cultural barriers within specific academic settings that center on the ‘publish or perish’ dogma, often leading to less data sharing, conservative views on intellectual property rights, time and resource pressures, and other impediments that slow transitions to open science workflows. Needless to say, no single entity or strategy can address all these challenges, and concerted efforts are required from researchers, funders, governments, and other stakeholders. However, by bringing together multiple, mutually informing projects meant to enable FAIR research to accelerate discovery in neuroscience, the CONP has contributed resources that aim to improve access to open science practices and aid researchers on multiple fronts.

Another key challenge has been to accurately abstract common needs across disparate research communities. The CONP has created and fostered an alliance of research institutions spanning many neuroscience research domains, regulatory jurisdictions, and institutional roadmaps and cultures. This process has unsurprisingly run up against entrenched habits, local interests and initiatives, and differing constraints issued by local governing bodies that sometimes appear to be at odds or in competition with what the alliance is trying to develop. For example, in what concerns data privacy, legal differences between Canadian provinces and how those legal guidelines are themselves interpreted by each local Research Ethics Board can sometimes give rise to important obstacles in trying to create harmonized policies for the open neuroscience community as a whole. The CONP strives to continue building trust and buy-in from the neuroscience community despite reluctance in some quarters to embrace some open science practices. Creating and communicating an incentive structure that is meaningful at multiple scales, from the individual researcher to the local department/institution, to national and international scales, will be critical in this next phase of operations for the CONP.

As is the case for all similar initiatives, the CONP must also develop the means for long-term sustainability, as even the successful founding of a platform and its provision of useful resources to the community does not itself guarantee its flourishing. One avenue that could be explored is the possibility of commercial partnerships, which have been demonstrated to play a key role in the open science movement by institutes such as the Structural Genomics Consortium. Many commercial entities in the research landscape see the value of open science practices in data reproducibility and publishing, and are willing partners and funders for open science research programs [54]. Additionally, many private science organizations have vast amounts of valuable data that has been used to answer a limited number of end-point questions and for which they have little further use. Making these openly available for broader exploration by the scientific community through partnerships would maximize their scientific potential and synergise both the potential pharmaceutical investment and that of other CONP funders.

## Outreach and impact on the community

The CONP’s objectives are ambitious and cover a gamut of topics crucial to the promotion of open science practice in the neurosciences. Many substantive challenges arise in the creation of new infrastructure, development of governance policy, raising of awareness and promotion, communication of incentive structure, and building a user-base that will eventually be large enough for members of the research community itself to have a motivating stake in the health, continued development, and future of the platform. Consequently, the CONP is still in its early phases, and metrics to measure success and impact in the neuroscience community are still being developed and implemented.

However, there are success stories that give insight to the functionality of this platform within the neuroscience community. One of CONP’s most highly accessed datasets (more than 3,000 total visits), from the **P**resymptomatic **E**valuation of **E**xperimental or **N**ovel **T**reatments for **A**lzheimer **D**isease (PREVENT-AD) cohort, is illustrative of the Portal’s flexibility. This is a longitudinal cohort study of presymptomatic Alzheimer’s disease patients containing deeply phenotyped, quality-controlled data, including MRI scans, CSF samples, and cognitive evaluations from more than 300 participants [23]. The open components of this dataset can be accessed directly, while the registered-access components can be accessed through the LORIS database interface or via the CONP Portal’s Datalad functionality once an account is obtained.

Another dataset that serves as an example of the impact the CONP seeks to have in the neuroscience community by having multiple open-neuroscience endeavors under the same roof is the open Calgary Campinas Brain MRI Dataset [55], which provides high-quality magnetic resonance imaging data of the human brain for a variety of important research applications. A CONP-supported scholar project led to the extension of its scientific utility for the community and the eventual native hosting of the dataset through the CONP Portal, which helped make its value that much more accessible, equitable, and fruitful. The Calgary Campinas Brain MRI Dataset has elicited very strong interest from the community and quickly became the most frequently accessed of the Portal’s collection, with nearly 4,000 views and over 400 downloads.

The CONP was built and is maintained by Canadian scientists but aims to operate for the benefit of a broader neuroscience community. Currently, submissions to the Portal are from research groups spanning over 15 institutions across Canada and 8 institutions outside of Canada. However, visits to the Portal from within Canada only account for approximately one third of the total, with the remainder spanning over 60 countries across 6 continents, indicating the potential utility of the CONP to a larger population of researchers. Although the majority of CONP capabilities can be used by any researcher, those requiring a Digital Research Alliance of Canada account is primarily restricted to Canadian scientists and their sponsored collaborators.

The CONP strives to build wider engagement and promote open science practices with researchers and other stakeholders in the neuroscience community and beyond by using a variety of different media, including conferences, webinars, podcasts, social media, and our website and newsletters. These CONP initiatives provide a platform for discussion and exploration of topics relating to open science and forward-looking academic practices with thought-leaders in the neuroscience, data sharing, and open science communities. CONP outreach efforts also focus on promoting the achievements of early career researchers, such as CONP scholars, as well as new datasets, pipelines, and notebooks shared by the broader community through the CONP. Submissions to the CONP, to the Portal, or to NeuroLibre, for example, may be featured in our “Research Spotlight” blog, with the aim of improving the profile of both the researcher and their research outputs. Similarly, short talks by CONP scholars have been shared through the various CONP outlets in our “Scholar Showcase.”

## Summary

The CONP has built an open neuroscience platform that includes (i) infrastructure for the sharing and use of data and tools, (ii) training resources and support for early career neuroscientists, (iii) ethics and policy frameworks for data governance and dissemination, (iv) an innovative preprint server for interactive and reproducible research reports, and (v) partnerships with like-minded international initiatives with similar open science principles. Moving forward, we aim to continue to expand the volume and breadth of data, tools, fully reproducible publications, and educational resources shared, accessed, and used through these platforms.

The CONP is developing stronger interoperability paradigms with other international data-sharing platforms (EBRAINS, NITRC, OpenNeuro, etc.) and is expanding its tool inventory (e.g., multivariate statistics, multimodal modeling, genomic analysis), with enhanced support for machine learning applications [2]. The NeuroLibre initiative has developed a platform for fully reproducible publications and worked with academic journals (*eLife*, *PLoS*) to prototype the use of Jupyter Notebooks for creating executable research articles. CONP training programs have directly supported the work of young, multidisciplinary neuroscience researchers at Canadian institutions to develop and openly share data, tools, and other resources with the community, as well as methods for curation and international dissemination of educational materials through the INCF. Ethics and data governance policies of general utility for open science initiatives have been shared with the community and CONP technologies support brain research networks across multiple technologies and research topics.

Although the neuroscience community is still working towards the wide-scale adoption of open science practices, the potential reward of attaining this goal for both for individuals and collectives of stakeholders is well worth the investment. As the drive to data and tool sharing becomes more broadly supported and required by public and philanthropic funding organizations, the CONP will continue to develop solutions that may help us achieve an optimal balance, all of which will be shared with the broader biomedical research and discovery community.
